# Fluorescence of ocular surface in a Covid -19 patient after Favipiravir treatment: a case report

**DOI:** 10.1186/s12985-021-01610-3

**Published:** 2021-07-13

**Authors:** Mehmet Ali Doran, Hasan Aytogan, Emre Ayıntap

**Affiliations:** Department of Ophthalmology, Tepecik Training and Research Hospital, Izmir, Turkey

**Keywords:** Fluorescence, Covid-19, Favipiravir, Ocular surface, Ultraviolet light

## Abstract

**Background:**

Favipiravir is used in treatment of Covid-19 patients. We aimed to share of ocular surface fluorescence in a patient after Favipiravir treatment in this case report.

**Case presentation:**

A 20-year-old male patient declared no known systemic disease prior to Covid-19. He applied to us with blurry vision and blue light reflection after Covid-19 treatment with Favipiravir. We observed bilateral fluorescence on his eyes and fluorescence of his nails. Biomicroscopic examination was insignificant.

**Conclusion:**

We investigated the fluorescence of favipiravir tablets under ultraviolet light. Drug demonstrated fluorescence. We recorded the favipiravir fluorescence in-vitro. This appears to be a strong evidence in terms of the linkage between the fluorescence of the ocular surface and favipiravir.

## Introduction

The WHO declared the coronavirus outbreak caused by the novel coronavirus designated as severe acute respiratory syndrome coronavirus 2 (SARS-CoV-2) that started at the end of 2019. The new coronavirus disease was termed as the “COVID-19” [1]. In a previous controlled trial, Favipiravir showed better treatment outcomes in COVID-19 patients in terms of disease progression and viral clearance [2]. The results of systematic review and meta-analysis showed that patients had clinical and radiological improvements following the treatment with Favipiravir [3]. Favipiravir is currently being used in treatment of Covid-19 patients in Turkey [4]. We aimed to share of ocular surface fluorescence in a patient after Favipiravir treatment in this case report.

## Case description

**Fig. 1:**
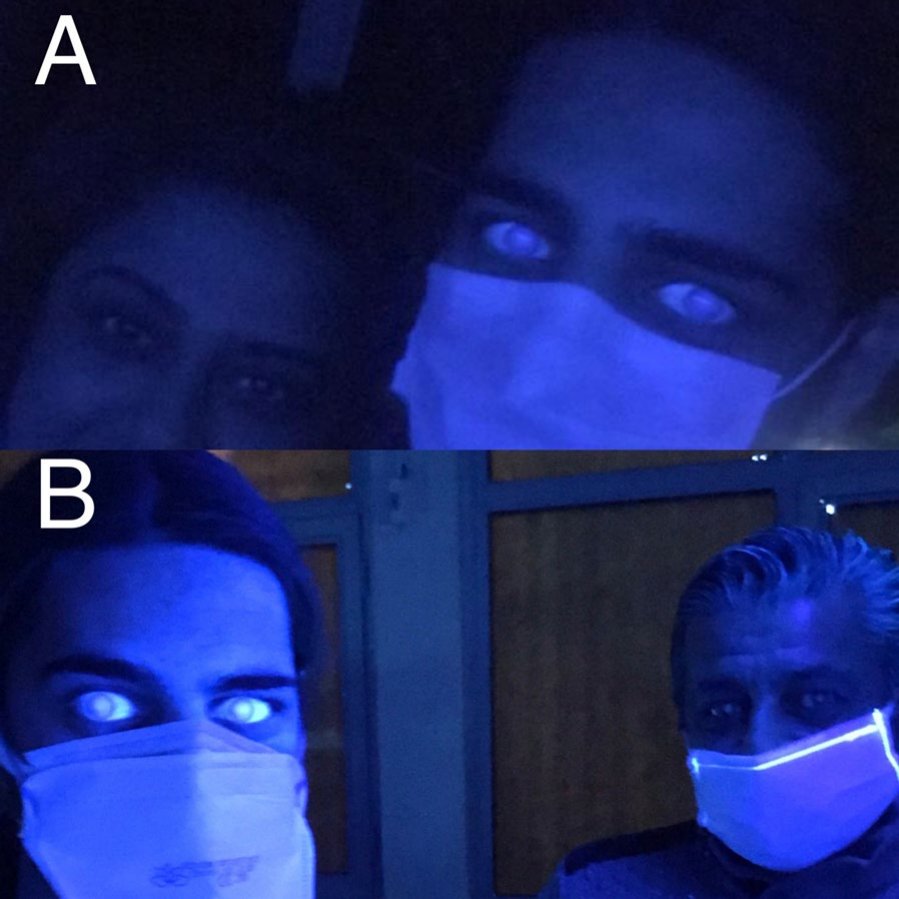
**A** Fluorescence of ocular surface after Favipiravir treatment in the patient (Right). Different appearance of patient’s eyes in the same environment with his mother under ultraviolet light. **B** Fluorescence of the patient’s eye in our dark examination room (Left). Different appearance of patient’s eyes in the same environment with his father under ultraviolet light

**Fig. 2:**
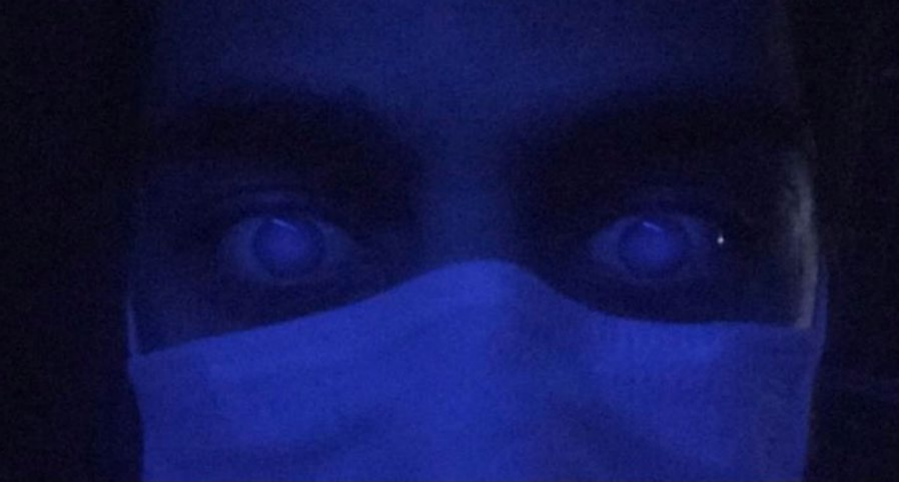
This photograph was taken in a dark room under UVL (365–395 nm wavelength) to demonstrate the bilateral fluorescence of eyes. Because of the fluorescence of Favipiravir, ocular surface of the patient behaves like a light source and demonstrates a flashing. Due to this very anterior fluorescence it is not possible to see a distinct pupillary reflection

**Fig. 3:**
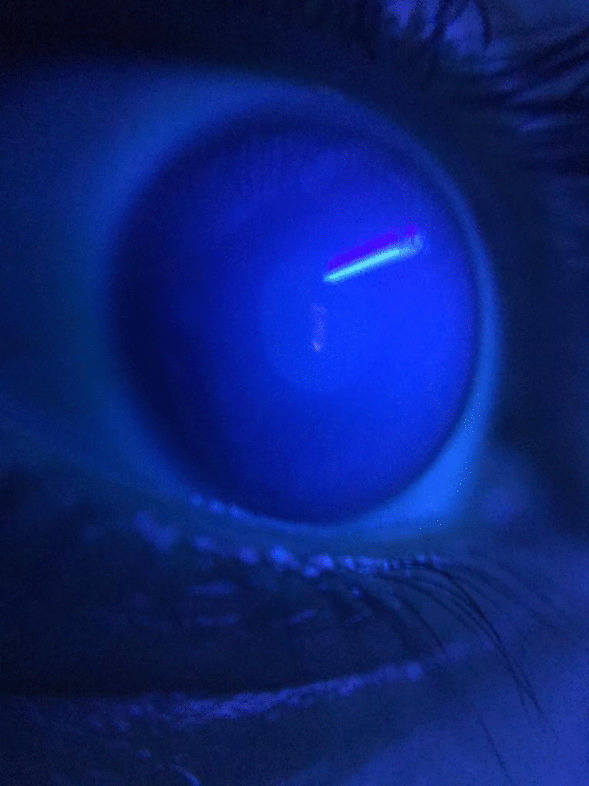
Close-up photograph of the patient's eye; Fluorescence on the entire ocular surface and ultraviolet light reflex in the central cornea. Barrier filters which were being used in anterior segment and fundus photography, over 365–395 nm were not used in this photograph to demonstrate the entire fluorescence of the ocular surface, this is why, anterior chamber and iris details are not visible in this magnified photogtaph

**Fig. 4:**
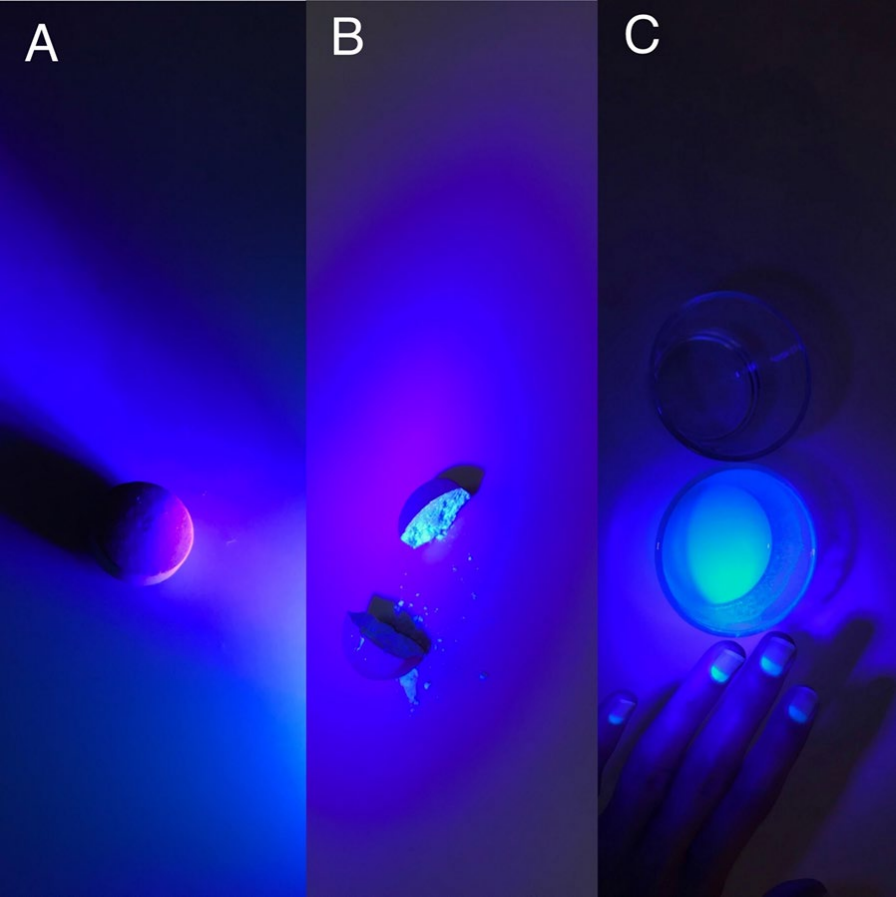
**A** Whole drug, **B** The drug in two halves, **C** Solved drug in water and patient’s nail fluorescence

**Fig. 5:**
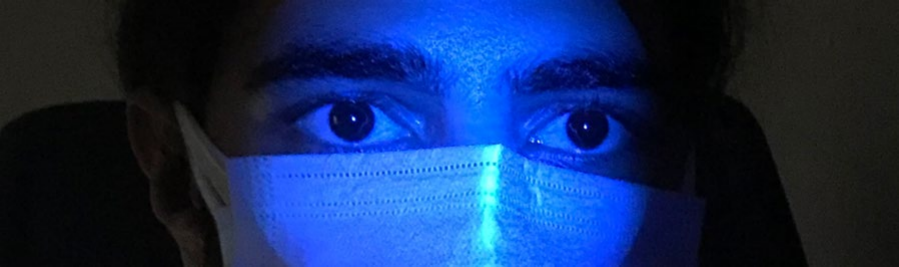
No fluorescence during the cobalt blue filter examination in slit lamp biomicroscopy (450–500 nm wavelength)

A 20-year-old male patient declared no known systemic disease prior to Covid-19. He applied to us with blurry vision and blue light reflection after Covid-19 treatment with Favipiravir. He was a photographer and had Ultraviolet light (UVL) sources in his room. These complaints occured only under UVL. A reverse-transcription-polymerase chain reaction test was positive for Covid-19 and he was at the second day of treatment of Favipiravir. Patient brought the photographs which was taken by himself under UVL. He took control photographs with his parents to determine that whether he was the only person affected from UVL. (Fig. 1A) We evaluated the photographs and close-up photo which were taken by himself. We observed bilateral fluorescence on his eyes (Figs. 2, 3). He brought ultraviolet light to the hospital because we wanted to make clear the patient's complaints. Patient’s UVL source had 365–395 nm wavelength which can be reffered as UV-A. Using the ultraviolet light source of the patient, we observed fluorescence of the patient’s eye in our dark examination room (windows covered with black films) (Fig. 1B). Favipiravir tablet was evaluated to demonstrate the fluorescence in-vitro (Fig. 4A, B). Patient had a nail fluorescence which was demonstrated in Fig. 4C. We examined the patient under cobalt blue filter which has 450–500 nm wavelength and we observed no fluorescence during the cobalt blue filter examination in slit lamp biomicroscopy (Fig. 5). Biomicroscopic examination was insignificant. His visual acuity was 20/20 bilaterally with Snellen Chart. Intraocular eye pressures were 16/17 mmhg. Anterior, posterior and angle optical coherence tomography findings were normal.

## Discussion

Favipiravir is currently being used in treatment of Covid-19 patients in Turkey [4]. It is recommended 2 × 1600 mg for the first day and 2 × 600 mg daily for the following four days. A recently published article demonstrated favipiravir induced nail and hair fluorescence [5]. In our literature search, we did not detect any case of ocular surface fluorescence. Here in, we reported the first case of ocular surface fluorescence in a patient under favipiravir treatment. In our examination, the patient did not show any fluorescence and visual impairment under room light and cobalt blue of a slit lamp which has 450–500 nm wavelength. Patient’s UVL source had 365–395 nm wavelength which can be reffered as UV-A. Ocular surface fluorescence disappeared after 14 days of favipiravir treatment but nail fluorescence was insisting.

Since the patient’s transient visual impairment was limited with the time which was meeting the duration of Favipiravir treatment, we did not attribute the visual impairement of the patient to the long-term outcomes of UVL contact. Because patient did not have any symptoms before or after the Favipiravir treatment. In addition, we have got four supportive reasons to accept that the flourescense of favipiravir prevented the patient's vision.

First, we may suggest that the timing of the complaints were complying with the beginning and the end of favipiravir treatment. If it is required to explain this suggestion, although patient is a photographer and working under UVL, patient strongly specified that he had never complained any transient or insisting visual impairement until he started to favipiravir treatment. Patient also reported that complaints were ceased after favipiravir treatment were stopped. In this regard, we may suggest that favipiravir treatment and complaints of the patient were consistent in terms of timing.

Second, while patient had visual impairement only during favipiravir treatment under UVL, parents were not taking Favipiravir treatment and this is why, patient had decided to test his parents as a control group in terms of whether they would experience same visual complaints or not. They noted that the patient was the only one who was complaining the fluorescence and visual impairment under the UVL.

Third, even if the anamnesis of the patient and all signs were indicating that the Favipiravir was responsible for the visual impairment, we wanted to prove the flourescense of favipiravir in an objective way with no intervention to the patient not to lead any damage. Therefore, we demonstrated the flourescence of the Favipiravir tablet in-vitro with the same UVL source of the patient had in his photography room. We investigated the fluorescence of favipiravir tablets under UVL with three different conditions. First, whole drug was photographed, second, we splitted the drug in two halves and third we solved the drug in water. Drug demonstrated fluorescence under second and third condition. Thus, we recorded the favipiravir fluorescence in-vitro (Fig. 4A–C).

Fourth, a recent study which was conducted by dermatologists reported that the hair and nail fluorescence in Covid-19 patients and they suggested that Favipiravir was the reason for fluorescence.^5^ Our patient had a nail fluorescence along with the ocular fluorescence.

## Conclusion

We investigated the fluorescence of favipiravir tablets under UVL. Drug demonstrated fluorescence in-vitro conditions. This appears to be a strong evidence in terms of the linkage between the fluorescence of the ocular surface and favipiravir.

## Data Availability

Not applicable.

## Abbreviation

UVL: Ultraviolet light
